# Effect of routine abdominal drainage on postoperative pain after uncomplicated laparoscopic cholecystectomy for cholelithiasis: A randomised controlled trial

**DOI:** 10.1016/j.amsu.2022.103353

**Published:** 2022-02-05

**Authors:** Farhad Fathi, Fereshteh Kamani, Ali Mohammad Farahmand, Shahab Rafieian, Matin Vahedi

**Affiliations:** a Department of Surgery, Taleghani Hospital, Faculty of Medicine, Shahid Beheshti University of Medical Sciences, Tehran, Iran; b Department of Thoracic Surgery, Imam Khomeini Hospital Complex, Tehran University of Medical Sciences, Tehran, Iran

**Keywords:** Laparoscopic cholecystectomy, Postoperative pain, Cholelithiasis, Drainage, Surgery

## Abstract

This is a prospective randomized controlled trial to investigate the effect of routine abdominal drainage on postoperative pain after uncomplicated laparoscopic cholecystectomy for cholelithiasis. This study was a single-center randomized controlled trial performed at the general surgery ward of Taleghani hospital, in Tehran, Iran, from July 2018 to October 2018. Patients were randomly divided into two parallel groups, one receiving routine abdominal drainage and the other receiving no treatment. Postoperative pain was measured by the Universal Pain Assessment Tool (UPAT) 0, 2, 4, 6, 12, and 24 h postoperatively. A total of 60 patients (30 patients in the study and control groups) were included. GLM repeated measure analysis showed a significant time*treatment effect for routine abdominal drainage in decreasing UPAT scores from baseline to 24 h after surgery (F = 4.59, df = 3.98, P-value = 0.001). Our findings demonstrated that abdominal drainage significantly reduces postoperative pain 0, 2, 4, 6, and 12 h after surgery (P-value<0.05). We also showed that abdominal drainage increases the time to first morphine sulfate administration and decreases the total dose of morphine sulfate administration (P-value<0.001). Moreover, we demonstrated that abdominal drainage decreases the average postoperative pain (P-value<0.001) and does not lead to any considerable side effects. However, 24 h after surgery, no significant pain-relieving effect was evident for abdominal drainage. In conclusion, insertion of abdominal drainage leads to decreased postoperative pain. Future studies need to investigate the optimal time for removal of the abdominal drain.

This trial was prospectively registered in the Iranian Registry of Clinical Trials with a registration ID of IRCT20130706013875N2.

## 1 Introduction

Postoperative pain is considered the leading cause of dissatisfaction, disability, and increasing complications after surgeries. The pain leads to recovery delay and postpones restoring regular physical activity after surgery. Different methods have been introduced for pain-relieving after surgical procedures [1]. Opioids are the most common postoperative analgesic agent, which causes various complications in patients, including exacerbation of ileus and bowel dysfunction after abdominal surgeries [2]. Thus, there is an increasing need for pain-relieving methods for postoperative pain, particularly in abdominal surgeries.

The standard surgical procedure for cholecystitis and cholelithiasis is laparoscopic cholecystectomy. Laparoscopic cholecystectomy is one of the most common gastrointestinal surgeries in general surgery [3]. During laparoscopic cholecystectomy, CO_2_ is often used to create space in the peritoneal cavity. Incomplete discharge of CO_2_ after the procedure stimulates the peritoneum and causes abdominal pain and also referral pain in the left shoulder [1,4].

Abdominal drainage has been vastly used in multiple abdominal surgeries for various objectives, including removing infected debris, preventing abscess formation, and healing leakage or fistula. Some surgeons use routine sub-hepatic drainage after laparoscopic cholecystectomy [5,6]. In recent studies, the use of routine administration of abdominal drainage after laparoscopic cholecystectomy has been limited to patients with intra-operative complications, including incomplete homeostasis and bile leakage [[7], [8], [9], [10]]. While in some other studies, drainage has been reported as a postoperative pain relief agent [[11], [12], [13]]. The current evidence is discrepant regarding the efficacy of abdominal drainage for relieving postoperative pain after laparoscopic cholecystectomy. This is a prospective randomized controlled trial to investigate the effect of routine abdominal drainage on postoperative pain after uncomplicated laparoscopic cholecystectomy for cholelithiasis.

## 2 Patients and methods

### 2.1 Trial design

This study was a single-center randomized controlled trial designed to investigate the effect of routine abdominal drainage on postoperative pain after uncomplicated laparoscopic cholecystectomy for cholelithiasis. It was performed at the general surgery ward of Taleghani hospital, in Tehran, Iran, from July 2018 to October 2018. Patients were randomly divided into two parallel groups, one receiving routine abdominal drainage and the other receiving no treatment. An informed consent statement was obtained from all patients before entering the trial. The ethics committee of Shahid Beheshti University of Medical Sciences approved the study with the number IR.SBMU.MSP.REC.1397.195. This trial is conducted and reported in line with the CONSORT criteria, and the entire checklist is submitted as an attachment (http://www.consort-statement.org/). The trial was conducted in accordance with the Declaration of Helsinki and its later revisions. This trial was registered in the Iranian Registry of Clinical Trials with a registration ID of IRCT20130706013875N2 (https://www.irct.ir/trial/34389).

### 2.2 Participants

Included participants were 18-75-year-old patients undergoing elective laparoscopic cholecystectomy for cholelithiasis. Exclusion criteria were 1) non-elective surgery, 2) ASA physical status more than 3, 3) history of opioid sensitivity, and 4) renal insufficiency and coagulopathy.

### 2.3 Procedure and interventions

The protocol for general anesthesia for all patients was the same. All patients were moved to the operation hall and monitored for blood pressure, oxygen saturation, pulse rate, capnometry, and ECG. All patients were premedicated with midazolam (2.5 mg) and fentanyl (2 μg/kg). Anesthesia was induced by thiopental Na (3–5 mg/kg) and atracurium (5 mg/kg). Anesthesia was maintained by isoflurane (0.8%–1.2%) in 50% N2O–O2 mixture. Before skin incision, 1 μg/kg of fentanyl was injected. Atracurium and fentanyl were administered as needed during anesthesia. After skin closure, neostigmine (40 μg/kg) and atropine (20 μg/kg) were administered to antagonize the remaining neuromuscular blockade. At the end of the procedure, pneumoperitoneum was discharged with low-vacuum suction. In the study group, a drain was inserted in the sub-hepatic region through the right upper quadrant trocar incision. In the control group, no drainage was inserted. All surgeries were carried out by an attending professor with the aid of assistants.

### 2.4 Outcomes and complications

After regaining consciousness, pain intensity was assessed using the Universal Pain Assessment Tool (UPAT) (time 0). This assessment was repeated subsequently at 2, 4, 6, 12, 24 h after surgery. The primary outcome measure of this study was pain intensity assessed with the Universal Pain Assessment Tool (UPAT) across study time points. Morphine was administered on a protocoled schedule based on patients’ demands. The time that patient demanded first analgesic medication, the total dose of morphine, and complications in the postoperative period were the secondary outcome measures of the study.

### 2.5 Sample size

Based on a pilot study, an effect size of 0.65 in the UPAT score at time 0 chest the two groups was presumed. Considering a power of 80% and a 2-tailed significance level of 5%, and an attrition rate of 10%, the sample size for each group was calculated as 30.

### 2.6 Randomization

The patients in this trial were randomized and allocated into two equal groups with a ratio of 1:1 and a block size of 4. A specific random code that was created by using Microsoft Office Excel was given to each patient. Patients, staff responsible for assessing the pain level and morphine dose postoperatively, and the statistician were not aware of the specific codes and intervention mode of each patient.

### 2.7 Statistical analysis

The Statistical Package of Social Science Software (SPSS version 20, IBM Company, USA) was used to analyze the data. Continuous variables are reported as mean ± SD, and categorical variables are demonstrated as a frequency using percentages. The two-tailed student T-test was used to compare two groups for continuous variables, when appropriate. Categorical variables were compared by implementing the Chi-squared test or Fisher's Exact test. General linear model (GLM) repeated measures analysis was applied to assess the time, treatment, and time*treatment effects. If Mauchly's test of sphericity was significant, a Greenhouse–Geisser adjustment in degrees of freedom was made. A P-value of <0.05 was considered statistically significant in all analyses.

## 3 Results

### 3.1 Demographic and baseline clinical data

**Fig. 1:**
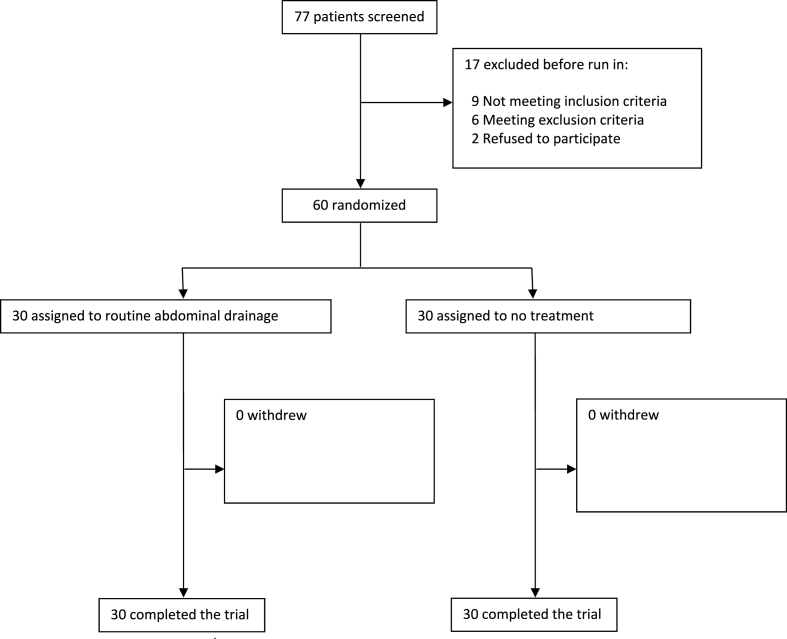
Flow diagram of trial.

A total of 77 candidates for laparoscopic cholecystectomy for cholelithiasis were screened against the inclusion and exclusion criteria and 60 patients were included and randomly allocated to two groups of routine abdominal drainage or control group in a 1:1 ratio (Fig. 1). No patient withdrew from the trial. A total of 60 patients (30 patients in the study and control groups) were included in this study. Demographic and baseline clinical data of patients after surgery in two trial groups are detailed in Table 1. Two trial groups were comparable based on age, gender, body mass index (BMI), cigarettes or opium consumption, SBP, DBP, and heart rate (P-value>0.05 for all). However, the duration of operation was significantly greater in the study compared to the control group (P-value<0.002).

**Table 1:** Baseline characteristics of the patients in two trial groups.

		Study group (n = 30)	Control group (n = 30)	P-value
Age (years; mean ± SD)		45.53 ± 13.11	49.00 ± 12.63	0.301
Gender (n (%))	Male	6 (20%)	10 (33.3%)	0.191
	Female	24 (80%)	20 (66.7%)	
BMI (kg/m^2^; mean ± SD)		28.95 ± 2.44	27.29 ± 3	0.08
Cigarettes or opium consumption (n (%))		6 (20%)	9 (30%)	0.371
SBP (mmHg; mean ± SD)		113.33 ± 13.47	115.67 ± 11.04	0.466
DBP (mmHg; mean ± SD)		72.00 ± 8.86	75.33 ± 7.76	0.127
Heart rate (beats/minutes; mean ± SD)		76.70 ± 4.85	78.20 ± 7.68	0.370
Duration of operation (minutes; mean ± SD)		55.83 ± 8.2	49.3 ± 7.51	**0.002**

BMI: Body Mass Index; SBP: Systolic Blood Pressure; DBP: Diastolic Blood Pressure.

P-value of <0.05 was considered statistically significant.

### 3.2 Postoperative pain

GLM repeated measure analysis showed a significant time*treatment effect for routine abdominal drainage in decreasing UPAT scores from baseline to 24 h after surgery (F = 4.59, df = 3.98, P-value = 0.001). Furthermore, separate time (F = 128.14, df = 3.98, P-value<0.001) and treatment (F = 17.39, df = 1, P-value<0.001) effects for routine abdominal drainage in decreasing UPAT scores from baseline to 24 h after surgery were also significant.

**Fig. 2:**
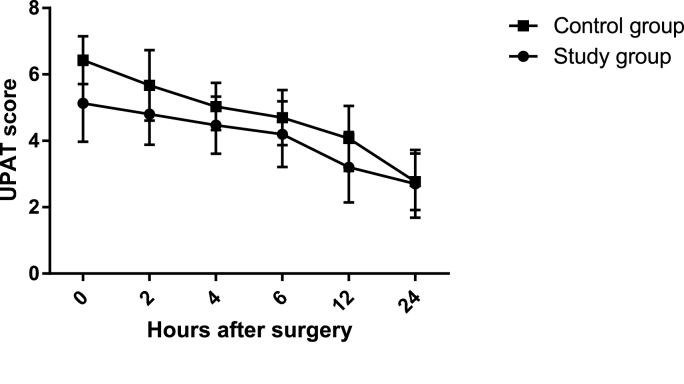
UPAT scores in two trial groups.

Table 2 and Fig. 2 illustrate the UPAT scores and change scores in different study time points after surgery. Compared to control group, patients in the study group had significantly lower UPAT scores 0 (P-value<0.001), 2 (P-value = 0.001), 4 (P-value = 0.008), 6 (P-value = 0.040), and 12 (P-value = 0.002) hours after surgery. However, no significant between-group difference was found 24 h after surgery. To control for the confounding effects of smoking and opium addiction, we repeated the analysis after excluding smokers and opium addicts (6 patients in the study and 9 patients in the control groups). The results remained the same in nonsmoker and non-addict patients (24 patients in the study and 21 patients in the control groups).

**Table 2:** UPAT scores in two trial groups.

UPAT scores		Study group (n = 30)	Control group (n = 30)	P value
Hours after surgery	0	5.13 ± 1.16	6.43 ± 0.72	**<0.001**
	2	4.8 ± 0.92	5.67 ± 1.06	**0.001**
	4	4.47 ± 0.86	5.03 ± 0.71	**0.008**
	6	4.20 ± 0.99	4.70 ± 0.83	**0.040**
	12	3.20 ± 1.06	4.07 ± 0.98	**0.002**
	24	2.70 ± 1.02	2.77 ± 0.85	0.785
Consecutive change scores	0–2	0.33 ± 0.88	0.76 ± 0.81	0.053
	2–4	0.33 ± 0.84	0.63 ± 0.80	0.165
	4–6	0.26 ± 1.04	0.33 ± 0.75	0.779
	6–12	1.00 ± 1.11	0.63 ± 0.99	0.185
	12–24	0.50 ± 0.97	1.30 ± 1.08	**0.004**
Change score from baseline	0–4	0.66 ± 0.99	1.40 ± 0.67	**0.001**
	0–6	0.93 ± 1.38	1.73 ± 0.98	**0.012**
	0–12	1.93 ± 1.38	2.36 ± 1.03	0.175
	0–24	2.43 ± 1.38	3.66 ± 1.09	**<0.001**
Average UPAT score		4.10 ± 0.71	4.81 ± 0.61	**<0.001**

P-value of <0.05 was considered statistically significant.

Compared to the control group, patients in the study group had significantly lower UPAT change scores between 12 and 24 h after surgery (P-value = 0.004) and between baseline with 4 (P-value = 0.001), 6 (P-value = 0.012), and 24 (P-value<0.001) hours after surgery. The results remained the same in nonsmoker and non-addict patients (24 patients in the study and 21 patients in the control groups).

Finally, compared to the control group, patients in the study group had significantly lower average postoperative UPAT scores (P-value<0.001). The results remained the same in nonsmoker and non-addict patients (24 patients in the study and 21 patients in the control groups).

### 3.3 Use of analgesics

As depicted in Table 3, the time to first analgesic administration was significantly increased in the study compared to the control group (P-value<0.001). Moreover, the total administered dose of morphine sulfate (MS) was significantly decreased in the study compared to the control group (P-value<0.001).

**Table 3:** Use of analgesics in two trial groups.

	Study group (n = 30)	Control group (n = 30)	P-value
Time to first analgesic administration (minutes; mean ± SD)	137.00 ± 94.76	48.67 ± 29.82	**<0.001**
Total MS dosage (mg; mean ± SD)	20.00 ± 8.61	30.47 ± 12.35	**<0.001**

MS: Morphine Sulfate.

P-value of <0.05 was considered statistically significant.

### 3.4 Complications

Table 4 illustrates the postoperative complications. Two trial groups were comparable based on complications. In the case of infection, only one of the patients suffered from brief cellulitis around the drainage site, which was identified in the follow-up visit. Moreover, there was no hemorrhage or bile drainage in the patients.

**Table 4:** Complications in two trial groups.

	Study group (n = 30)	Control group (n = 30)	P-value
Nausea (n (%))	22 (73.3%)	19 (63.3%)	0.405
dizziness (n (%))	15 (50.0%)	13 (43.3%)	0.605
Somnolence (n (%))	19 (63.3%)	17 (56.7%)	0.598

P-value of <0.05 was considered statistically significant.

## 4 Discussion

Cholecystectomy is the second most common surgical procedure in the gastrointestinal tract after appendectomy. Traditionally, some surgeons use routine prophylactic drainage after laparoscopic cholecystectomy, but most recent studies have reported their benefits in vain [3,4]. In this study, the effect of sub-hepatic drainage on postoperative pain was evaluated. A systematic review with meta-analysis and sequential trial analysis by Yong et al. indicates a significant decrease in the severity of pain when drainage was not used [14]. In the present study, the use of drain significantly reduced the postoperative pain at 0 h (immediately after surgery and at the entrance to the ward), 2, 4, 6, and 12 h after the surgical procedure. However, our results showed more significant pain decreased in the control compared study group, which is probably due to higher levels of MS administration in patients in the control group. Our findings demonstrated that abdominal drainage increases the time to first MS administration and decreases the total dose of MS administration. Moreover, we demonstrated that abdominal drainage decreases the average postoperative pain and does not lead to any considerable side effects.

The morphine dose in this study in the group with drain was lower than that of the other group, which was similar to the report of Nursal et al. [11], although their results did not have a significant difference. Other studies did not show a significant difference in the reduction of analgesic use compared to different groups [12].

All of our patients were discharged on the day after surgery. In some studies, the use of drain has been suggested as a factor for the later discharge of patients and hence longer hospital stay [[15], [16], [17]]. However, some studies have not indicated a change in the patient's discharge time [18].

Although we showed less pain severity in the drain group at the first hours after surgery, the pain intensity 24 h after surgery was comparable between the study and the control group. This might be justified because over time and probably due to the sealing of the drain with omentum, its function decreases [15], and the drain might lead to irritation of the skin and peritoneum, especially after the physical activity of the patients. These cause exacerbation of the pain in the drain group in 24 h following surgery.

Other studies evaluated the amount of postoperative fluid collection by ultrasonography and analyzing the pain severity-fluid collection correlation after the first day; meanwhile, our study focused on the pain severity in the first hours after surgery without measurement of postoperative fluid collection. Furthermore, in our study, the patients with drain had the first dose of analgesia later than those in the control group. In other studies, the time of administration of the first dose of the analgesia has not been investigated. On the other hand, patients who received the first dose of the analgesia earlier received a higher dose of the drug during the 24 h after the operation.

In our study, the duration of surgery in patients with drainage was about 6 min longer than that of the control group, which was similar to the Picchio et al. [15], which lasted about 7 min longer, and the Yong et al. [14], and the study Uchiyama et al. [9] which lasted 5.77 min and 1 min more than the study group. However, the longer duration of the operation in the drain group is negligible.

The effect of subhepatic drainage on postoperative pain in patients who underwent laparoscopic cholecystectomy has been investigated by previous studies [13]. In the majority of previous studies, subhepatic drainage had no effect on postoperative pain after laparoscopic cholecystectomy [15,16,18,19], which is inconsistent with our findings. Nonetheless, in most of the previous studies, postoperative pain was assessed 24 h after surgery. Meanwhile, our study emphasizes the pain severity in the first hours after the operation. Hence our results cannot be rendered to the whole postoperative time. Another reason for contradictory results might be the location of the drain. In our study, a drain was placed in the subhepatic region under the gallbladder bed. In contrast, in the study of Jorgensen et al. [20], the drain was located in the supra-hepatic area and only for the drainage of pneumoperitoneum.

One of the theories presented in the post-laparoscopic surgery pain is incomplete gas drainage and remaining pneumoperitoneum, which causes shoulder and abdominal pain [20]. In the study of Gurusamy et al. [12], suction drains have been shown to reduce pain in the postoperative period compared to passive drains. Moreover, Jorgensen et al. declared that the use of a passive suction drain in laparoscopic cholecystectomy decreases shoulder tip pain by allowing CO2 gas to escape the peritoneal cavity [20]. These findings are consistent with our results showing less severe pain in the drain group on the first day after the operation.

Irritation of the peritoneum due to gas can lead to nausea and vomiting. Despite a decrease in this side effect in the drain group, there was no significant difference between the trial groups. Chauhan et al. showed that active aspiration of CO2 after laparoscopic cholecystectomy greatly reduces postoperative nausea/vomiting and shoulder tip pain [16]. Headache and dizziness as an anesthetic and analgesic side effects were not significantly different between the two groups. These findings are similar to the findings of most other studies [12,15,16,18,19].

Our trial is not without limitations. The small sample size of patients is the major limitation of this study, which limits the generalizability of our findings. Future studies with larger sample sizes are needed to confirm these findings. Furthermore, a relatively short study duration might not disclose the long-term effects of the intervention. Multi-centered and long-term trials are required in this regard.

## 5 Conclusion

In conclusion, the effect of abdominal drainage on postoperative pain after laparoscopic cholecystectomy was investigated. Our findings demonstrated that abdominal drainage significantly reduces postoperative pain 0, 2, 4, 6, and 12 h after the surgical procedure. We also showed that abdominal drainage increases the time to first MS administration and decreases the total dose of morphine sulfate administration. Moreover, we demonstrated that abdominal drainage decreases the average postoperative pain and does not lead to any considerable side effects. However, 24 h after surgery, no significant pain-relieving effect was evident for abdominal drainage. Based on these, we suggest the insertion of abdominal drainage for decreasing postoperative pain; however, it is recommended that the drain get removed as soon as possible due to increased pain on the day after the operation. Future studies need to investigate the optimal time for removal of the abdominal drain.

## Provenance and peer review

Not commissioned, externally peer-reviewed.

## Ethical approval

Iran National Committee for Ethics in Biomedical Research organization of the Medical Ethics Committee with ethics code: IR.SBMU.MSP.REC.1397.195.

## Sources of funding

No.

## Author contribution

Author contribution FF: conception and design of the study, drafting the article, and approval of the final version of manuscript; FK: conception and design of the study, drafting the article, and approval of the final version of manuscript; AMF: acquisition of data, critical revise, and approval of the final version of manuscript; SR: acquisition of data, critical revise, and approval of the final version of manuscript; MV: conception and design of the study, drafting the article, analysis and interpretation of data, and approval of the final version of manuscript.

## Registration of research studies

1. Name of the registry: Iranian Registry of Clinical Trials
2. Unique Identifying number or registration ID: IRCT20130706013875N2
3. Hyperlink to your specific registration (must be publicly accessible and will be checked):https://www.irct.ir/trial/34389

## Guarantor

Matin Vahedi MD.

## Consent

“Written informed consent was obtained from the patient for publication of this case report and accompanying images. A copy of the written consent is available for review by the Editor-in-Chief of this journal on request”.

## Declaration of competing interest

No.
